# HDAC6 inhibition by tubastatin A is protective against oxidative stress in a photoreceptor cell line and restores visual function in a zebrafish model of inherited blindness

**DOI:** 10.1038/cddis.2017.415

**Published:** 2017-08-31

**Authors:** Janina Leyk, Conor Daly, Ulrike Janssen-Bienhold, Breandán N Kennedy, Christiane Richter-Landsberg

**Affiliations:** 1 Department of Neuroscience, Molecular Neurobiology, University of Oldenburg, Oldenburg D-26111, Germany; 2 School of Biomolecular and Biomedical Science, Conway Institute, University College Dublin, Belfield, Dublin D04 V1W8, Ireland; 3 Department of Neuroscience, Visual Neuroscience, University of Oldenburg, Oldenburg D-26111, Germany

## Abstract

Retinal diseases, such as hereditary retinitis pigmentosa and age-related macular degeneration, are characterized by the progressive loss of photoreceptors. Histone deacetylase 6 (HDAC6) is considered as a stress surveillance factor and a potential target for neuroprotection and regeneration. Overexpression of HDAC6 has been connected to neurodegenerative disorders, and its suppression may provide protection. Here we show that HDAC6 is constitutively present in the mouse retina, and in the cone-like mouse cell line 661W. In 661W cells HDAC6 inhibition by the specific inhibitor tubastatin A (TST) led to the acetylation of *α*-tubulin, which is a major substrate for HDAC6. After oxidative stress, exerted by hydrogen peroxide, TST promoted cell survival and the upregulation of heat-shock proteins HSP70 and HSP25 by activation of heat-shock transcription factor 1. Furthermore, in response to oxidative stress the redox regulatory protein peroxiredoxin 1 (Prx1) was modulated in 661W cells by HDAC6 inhibition. The peroxide reducing activity of Prx1 is dependent on its acetylation, which is mediated by HDAC6. Pre-incubation with TST prevented the inactivation of Prx1 and its preserved activity may exert protective effects in photoreceptor cells. To determine whether TST treatment has a therapeutic effect on visual function, the *dye*^*ucd6*^ zebrafish model of inherited sight loss was utilized. Zebrafish have developed as a suitable model system for pharmacological testing. *In vivo* application of TST caused the hyperacetylation of *α*-tubulin, indicating that HDAC6 is active in this model. Furthermore, TST was sufficient to rescue visual function and retinal morphology. Hence, HDAC6 inhibition and the regulation of peroxiredoxin activity may play a significant role in protecting retinal cells and in particular photoreceptors, which are exposed to high levels of reactive oxygen species derived from oxidative stress-induced injuries.

Blinding conditions, such as retinitis pigmentosa (RP), age-related macular degeneration and cone dystrophies (COD), are characterized by the progressive loss of photoreceptors. Despite the knowledge of causative mutations in 67 genes for RP and 25 genes for COD (RetNet: https://sph.uth.edu/retnet, date last accessed June 2017) no effective therapy is yet available. In RP, primary rod degeneration is followed by secondary cone cell death, and in COD cone photoreceptors are the major cells affected and subsequently degenerate.^1, 2^ The molecular mechanisms leading to cone degeneration, however, are still unclear and need elucidation.

Oxidative stress, which is defined as an imbalance between the level of reactive oxygen species (ROS) and antioxidant defenses,^3^ is connected to retinal degeneration. Since cone photoreceptors have a high metabolic demand and are characterized by a high content of unsaturated lipids, they are particularly susceptible to ROS-induced cell damage.^4, 5^ Increasing evidence obtained from animal RP models as well as from analyses of aqueous humor from RP patients suggests that oxidative stress contributes to secondary cone death in RP pathogenesis.^6, 7^ Furthermore, a variety of studies implicate that the application of antioxidants slows photoreceptor degeneration and promotes long-term survival of cones in mouse models of RP.^8, 9, 10^

In response to cellular stress situations, heat-shock proteins (HSPs) are induced.^11, 12^ HSPs are highly conserved proteins that act as molecular chaperones and as a first line of defense against cellular stress; they are induced by transcription factors, such as heat-shock factor 1 (HSF1).^13^ Recent studies suggest a protective role for HSPs in degenerative processes of the retina. Parfitt *et al.*^14^ and Aguila *et al.*^15^ demonstrated that induction of a heat-shock response delayed degeneration of photoreceptors and restored visual function in the P23H rat model for RP. Furthermore, heat-shock protein 70 (HSP70) induction may prevent photoreceptor cell death in rodent models of RP.^16^ Ischemic insults, oxidative stress and other factors induce the small heat-shock protein 27 (HSP27/HspB1) in retinal cells, providing neuroprotection due to its molecular chaperone activity, interaction with the cytoskeleton and prevention of apoptotic cell death.^17^

Recently a role for histone deacetylases (HDACs) in retinal stress responses and degeneration emerged.^18, 19, 20^ Mammalian HDACs are divided into four classes (HDAC I–IV) according to their homology to yeast HDACs. Class I, II and IV HDACs are zinc-dependent and structurally different from the NAD^+^-dependent class III HDACs, also known as sirtuins.^21^ Excessive activation of class I and II HDACs was observed in the *rd1* mouse model of RP as well as in the *cpfl1* mouse model of progressive cone dystrophy. Interestingly, inhibition of HDAC I/II activity, using the pan-inhibitor trichostatin A (TSA), prevented photoreceptor cell death in *rd1* organotypic retinal explants and *in vivo* protected cone photoreceptor cells in *cpfl1* mice.^18, 20, 22^

Thus, HDAC inhibitors have therapeutic potential in retinal degeneration. However, the molecular mechanisms underlying the protective effects of HDAC pan-inhibitors are not yet resolved. In particular, the question whether individual isoforms have beneficial effects has not been addressed. In this respect, HDAC6 is of special interest. Among the 18 members of the HDAC family, HDAC6 is a unique class II deacetylase. It possesses two catalytic domains and deacetylates mainly non-histone proteins, such as *α*-tubulin, cortactin and HSP90 (for recent reviews see refs 21, 23, 24). Furthermore, the redox regulatory protein peroxiredoxin 1, which is crucial for reducing hydrogen peroxide (H_2_O_2_), is a target of HDAC6.^25^ Additionally, HDAC6 is connected to activation of HSP genes^26^ and modulates cellular defense mechanisms.^24, 25, 27^

In the present study, we used the specific HDAC6 inhibitor tubastatin A (TST)^28^ to evaluate whether inhibition of HDAC6 can provide a protective means against oxidative stress exerted by H_2_O_2._ To better understand the molecular mechanisms elicited by TST on a cellular level, 661W cells, representing a mouse cone photoreceptor-like cell line,^29^ were used. To determine whether TST has a therapeutic effect on visual function and retinal morphology *in vivo*, the *dye*^*ucd6*^ (dying on edge) zebrafish model of inherited blindness, representing a cone dominant visual system, was used. The data show that HDAC6 inhibition plays an important role in neuroprotection.

## Results

### HDAC6 is expressed and active in retinal cells

**Figure 1:**
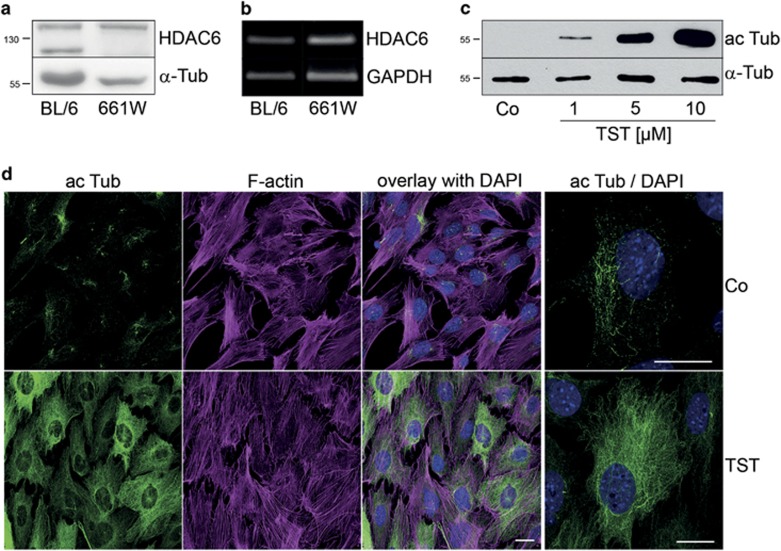
HDAC6 is expressed in mouse retinae and its inhibition by tubastatin A leads to hyperacetylation of *α*-tubulin in 661W cells. Immunoblot analysis (**a**) and RT-PCR analysis (**b**) revealed HDAC6 expression in lysates from C57BL/6J mouse retinae (BL/6) and 661W cells. *α*-Tub, *α*-tubulin. (**c**) Immunoblot analysis of 661W cell extracts incubated with tubastatin A (1, 5 and 10 *μ*M) for 24 h was carried out using antibodies against *α*-tubulin (*α*-Tub) and acetylated tubulin (ac Tub). (**d**) 661W cells were treated with 10 *μ*M tubastatin A (TST) for 24 h. Indirect immunofluorescence was carried out using an antibody against acetylated tubulin (ac Tub) and Phalloidin-CF594 was used to label F-actin. Nuclei were stained with DAPI. Co, untreated control. High magnification images of 661W cells revealed a pronounced increase of acetylated α-tubulin in TST-treated cells. Scale bars: 20 *μ*m

Mouse-derived cone-like 661W cells were used as a cellular model. To investigate whether HDAC6 transcript and protein is present in these cells, and to also verify its constitutive expression in the mouse retina, lysates from wild-type C57BL/6J mouse retinae and 661W cells were subjected to immunoblot analysis or reverse transcription polymerase chain reaction (RT-PCR). As shown in Figure 1, HDAC6 protein (Figure 1a) and mRNA (Figure 1b) is detected in retinal extracts and 661W cells. The specificity of the antibody against HDAC6 was verified by using a blocking peptide provided by the manufacturer (see Supplementary Information to Figure 1). To study whether HDAC6 is active in 661W cells and that cells react to its inhibition, cells were incubated with TST for 24 h and hyperacetylation of *α*-tubulin was assessed by immunoblot procedures and indirect immunofluorescence. Immunoblot analysis pointed to a concentration-dependent effect of TST to increase *α*-tubulin acetylation (ac Tub), which was prominent at a concentration of 10 *μ*M (Figure 1c). At even higher concentrations this was more pronounced; however, TST caused a slight cytotoxic effect. Hence, a concentration of 10 *μ*M TST was used for subsequent experiments. For indirect immunofluorescence an antibody against ac Tub was used, and to label F-actin Phalloidin-CF594 (red) was applied. In control cells ac Tub was only scarcely present and mainly the microtubule organizing center was prominently stained (Figure 1d). After incubation with 10 *μ*M TST microtubules were acetylated throughout the cell cytoplasm and cellular extensions, while the actin network seemed to be unaffected. However, cells appeared more adherent and flattened.

### TST protects 661W cells against oxidative stress exerted by H_2_O_2_

**Figure 2:**
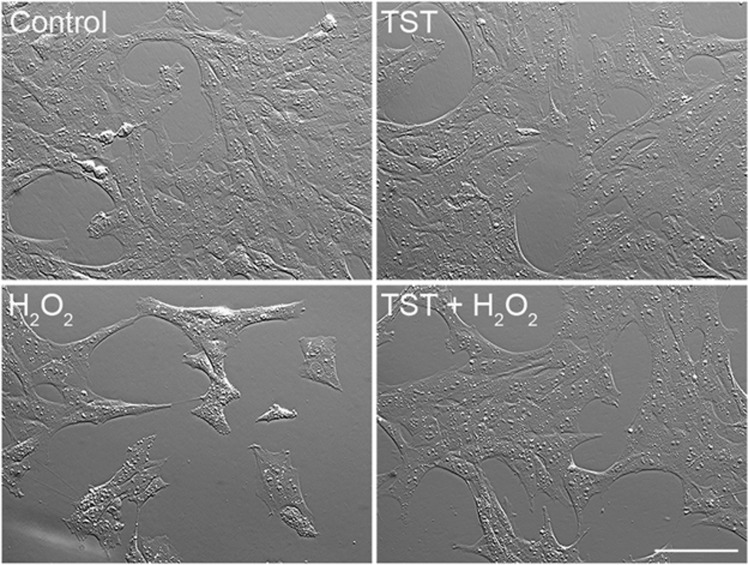
Pre-incubation with tubastatin A protects 661W cells against oxidative stress. Hoffmann modulation contrast images are shown. 661W cells were treated with 10 *μ*M tubastatin A (TST) for 8 h, with 200 *μ*M H_2_O_2_ for 6 h, or were preincubated with 10 *μ*M TST for 2 h followed by incubation with H_2_O_2_ for 6 h. Co, untreated control (*n*=5). Scale bar: 100 *μ*m

**Figure 3:**
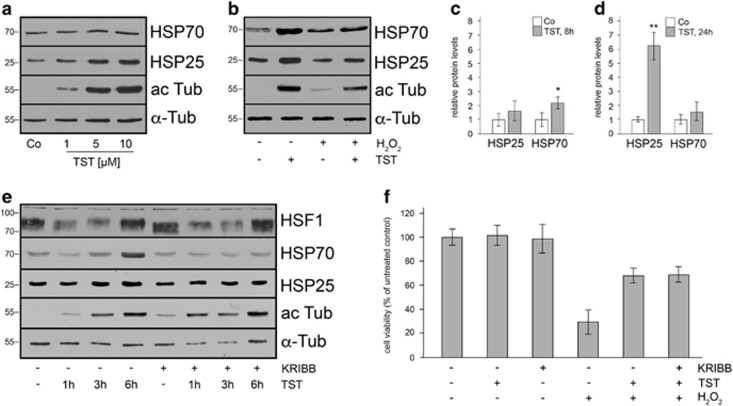
Tubastatin A induces heat-shock protein expression by activating heat-shock factor 1. 661W cells were treated with 1, 5 and 10 *μ*M of tubastatin A (TST) for 24 h (**a**), or in (**b**) with 10 *μ*M TST for 8 h, or with 200 *μ*M H_2_O_2_ for 6 h or were preincubated with 10 *μ*M TST for 2 h followed by incubation with 200 *μ*M H_2_O_2_ for 6 h. ac Tub, acetylated tubulin. *α*-Tub, *α*-tubulin. Co, untreated control. Quantitative evaluation of immunoblot analysis revealed a significant increase in heat-shock protein (HSP) 70 level after 8 h (**c**), while HSP25 was significantly enhanced after 24 h (**d**); *n*=4. (**e**) Heat-shock factor 1 (HSF1) activity was investigated using immunoblot analysis of 661W cell extracts that were treated 10 *μ*M TST for 1, 3 and 6 h, or with 5 *μ*M KRIBB11 (KR) for 6.5 h alone, or preincubated with 5 *μ*M KR for 30 min, followed by incubation with 10 *μ*M TST for 1–6 h. (**f**) Cell viability MTT assay. Cells were treated as indicated. TST (10 *μ*M) for 8 h or KR (5 *μ*M) for 8.5 h did not influence 661W cell number. H_2_O_2_ (200 *μ*M) for 6 h led to a strong decrease in cell viability, which was enhanced by pre-incubation with TST for 2 h (TST+H_2_O_2_). Pre-incubation with KR for 30 min followed by incubation with TST for 2 h followed by treatment with H_2_O_2_ for 6 h (KR+TST+H_2_O_2_) did not diminish the protective effect of TST. Experiments were carried out three times with similar results. Data represent the mean±S.D. of one representative experiment with eight replicates and are expressed as the percent of the untreated control, which was set at 100%

Since oxidative stress has been connected to retinal degeneration, we tested whether TST is protective and ameliorates the cytotoxic effects of oxidative damage in 661W cells. Towards this, 661W cells were incubated with 200 *μ*M H_2_O_2_ for 6 h either alone or after a 2 h pre-incubation with TST (10 *μ*M) followed by 200 *μ*M H_2_O_2_ for 6 h in the presence of TST. The microscopical images indicate that after treatment with H_2_O_2_ cells were severely damaged, cellular processes were retracted and less dense (Figure 2). Pretreatment with TST rescued the cells; they kept their healthy morphology and cell density was enhanced. Treatment with TST alone (8 h, 10 *μ*M) did not exert any cytotoxic effects. Hence, HDAC6 inhibition by TST exerted protective effects against oxidative stress in 661W cells, which was further corroborated by an MTT cell viability assay (see below, Figure 3).

### TST elicits a heat-shock response in 661W cells and causes the upregulation of HSP25 and HSP70

To investigate further the molecular events underlying the protective effects of TST, we evaluated whether TST promotes the induction of HSPs. Immunoblot analysis revealed that TST led to a significant upregulation of HSP25 (mouse homolog to human HSP27) and only a slight increase of HSP70 after 24 h in a concentration-dependent manner (Figures 3a and d). After 8 h of treatment with TST, HSP70 was significantly and HSP25 only slightly enhanced, as compared with the untreated control (Figures 3b and c). The induction of HSP25 and HSP70 was suppressed in the presence of H_2_O_2_ (Figure 3b).

To test whether TST-mediated HSP induction was accompanied by the activation of heat-shock transcription factor 1 (HSF1) and whether the inhibition of HSF1 activation may enhance the cytotoxic effects of H_2_O_2_, a specific inhibitor of HSF1, namely KRIBB11, was used.^30^ 661W cells were treated with TST (1, 3 and 6 h), or with 5 *μ*M KRIBB11 for 6.5 h, or preincubated with KRIBB11 for 30 min followed by TST treatment in the presence of KRIBB11 for further 1, 3 and 6 h. Cell lysates were prepared and subjected to immunoblot analysis using antibodies against HSF1 and as indicated. As seen in Figure 3e after 3–6 h of treatment with TST the migration velocity of HSF1 was slightly decreased and the protein appeared with a higher molecular weight, indicating its phosphorylation and activation. After the combined treatment with KRIBB11 and TST this small shift in migration was still observed, which is due to the fact that KRIBB11 inhibits only the recruitment of the positive transcription elongation factor b (p-TEFb) to the *hsp70* promoter and not the phosphorylation of HSF1.^30^ After treatment with KRIBB11 and TST the induction of HSP25 and HSP70 was suppressed as compared with the treatment with TST alone (Figure 3b), corroborating its activity.

Next, using an MTT assay, cell viability was assessed. Cells were treated either with TST or KRIBB11 alone, or with H_2_O_2_ alone and in combination with TST and KRIBB11 as described above. While TST and KRIBB11 did not exert cytotoxic effects, treatment with H_2_O_2_ caused a severe decrease in cell viability and less than 30% of the cells survived (Figure 3f). Pre-incubation with TST for 2 h followed by 6 h of incubation with 200 *μ*M H_2_O_2_ in the presence of TST (TST+H_2_O_2_) resulted in a significant enhancement of cell viability (68±6%), when compared with H_2_O_2_ treatment alone. Pre-incubation with KRIBB11 followed by TST and H_2_O_2_ did not affect cell viability as compared with the combined treatment with TST and H_2_O_2_ alone (Figure 3f). Hence, the inhibition of HSF1 and the heat-shock response were not sufficient to prevent the protective effects of TST on cell viability, suggesting that other factors might contribute.

### The activity of peroxiredoxin 1 is modulated by TST in response to oxidative stress

**Figure 4:**
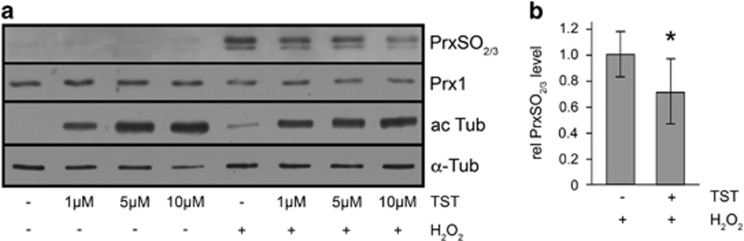
Pre-incubation with tubastatin A regulates the activity of peroxiredoxin 1. 661W cells were treated with tubastatin A (TST) for 8 h as indicated, or with H_2_O_2_ (200 *μ*M) for 6 h, and they were preincubated with 1, 5 and 10 *μ*M TST for 2 h followed by incubation with H_2_O_2_ for 6 h (TST+H_2_O_2_). (**a**) Immunoblot analysis of cell extracts revealed an H_2_O_2_ induced increase in the level of PrxSO_2/3_, the inactivated form of peroxiredoxin 1 (Prx1), which was significantly reduced by pre-incubation with 10 *μ*M TST, as shown by quantitative evaluation of the immunoblot (**b**). ac Tub, acetylated *α*-tubulin; *α*-Tub, *α*-tubulin. *n*=4

This prompted us to investigate whether TST in 661W cells has an influence on peroxiredoxin 1 (Prx1), which is involved in the reduction of H_2_O_2_ and has been identified as a substrate of HDAC6.^25^ Oxidative stress or H_2_O_2_ can lead to the overoxidation and inactivation of Prx1, which includes the increased formation of inactive overoxidized PrxSO_2/3_ molecules.^31^ 661W cells were incubated with TST (1, 5 and 10 *μ*M) alone for 8 h, or with 200 *μ*M H_2_O_2_ alone for 6 h, or were preincubated with increasing concentrations of TST for 2 h followed by 6 h of incubation with H_2_O_2_ (200 *μ*M) in the presence of TST. Cell lysates were subjected to immunoblot analysis using antibodies against *α*-tubulin, acetylated tubulin, Prx1 and PrxSO_2/3_, the overoxidized and thereby inactivated form of Prx1. Anti-Prx1 antibodies recognize Prx1 independent of its oxidation. Figure 4 demonstrates that H_2_O_2_ promoted the overoxidation of Prx1 as indicated by the increased level of PrxSO_2/3_. Pre-incubation with TST in a concentration-dependent manner resulted in decreasing levels of PrxSO_2/3_, while the levels of Prx1 remained at the same level. Thus, TST treatment prevented the inactivation of Prx1, and its preserved activity may exert protective effects.

### TST restores visual function and retinal morphology in a zebrafish model of inherited blindness

Zebrafish have been increasingly used as a system to model neurodegeneration and for the study of human eye disorders, since it has similarities to the mammalian nervous system, and has emerged as a vertebrate model organism to combine *in vitro* and *in vivo* assays.^32, 33^ Previous data confirm that in zebrafish, *hdac6* is expressed in the larval eye during maturation of visual function,^19^ and specifically in cone photoreceptors as determined by microarray analysis of cone-enriched transcripts from adult zebrafish.^34^ The data are deposited in the NCBI Gene Expression Omnibus (GEO) database, under accession number GSE86155. Furthermore, in a recent study Pinho *et al.*^35^ have demonstrated that zebrafish HDAC6 shows high sequence identity with the mammalian homolog at its deacetylase active site. In their study the effects of HDAC inhibitors on a zebrafish model of Parkinson’s disease were evaluated. In this model TST increased the levels of acetylated α-tubulin, could not repair 1-methyl-4-phenyl-1,2,3,6-tetrahydropyridine (MPP^+^)-induced locomotor impairments but could rescue metabolic activity in whole larvae.^35^

**Figure 5:**
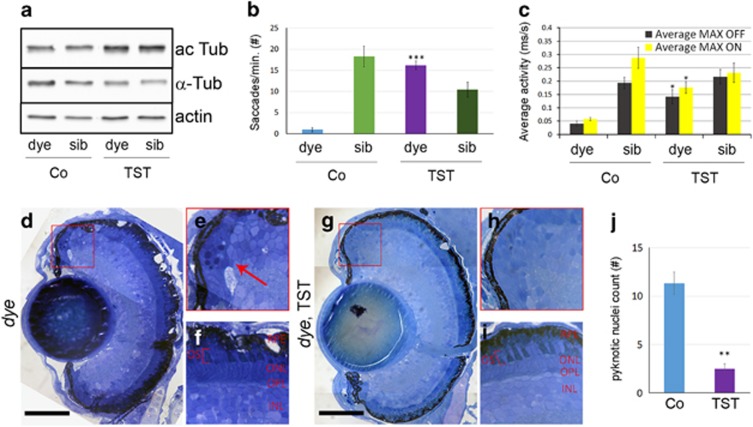
Tubastatin A treatment rescues visual function and retinal morphology defects in the *dye* zebrafish model of inherited blindness. (**a**) *Dye* mutants and siblings were treated with 100 *μ*M tubastatin A (TST) from 3 to 5 dpf, lysates were prepared from 50 larval eyes each and subjected to immunoblot analysis using antibodies against *α*-tubulin (*α*-Tub), acetylated *α*-tubulin (ac Tub) and actin as a loading control, *n*=3. (**b**) *dye* mutants treated with TST revealed significantly improved OKR when compared with vehicle (0.1% DMSO)-treated mutants, error bars show standard error of the mean. (**c**) Summary of VMR activities. Treatment with 100 *μ*M TST significantly improves the MAX OFF (black bars) and MAX ON (yellow bars) activities in *dye* mutants. For visual function data, *N*=12 (number of larvae per replicate), *n*=3 (number of replicates), error bars show standard error of the mean. (**d**–**i**) Light micrographs of retinal sections derived from TST (**g**–**i**) and vehicle-treated *dye* larvae (**d**–**f**). Treatment with 100 *μ*M TST reduces the number of dying cells in the ciliary marginal zone (red square in **g** and **h**) indicated by the presence of pyknotic nuclei compared with control larvae (red square in **d**, red arrow in **e**), scale bars represent 100 μm. The number of pyknotic nuclei present in multiple sections through the central retina is also reduced (bar chart in **j**). Photoreceptor outer segment length is marginally improved following TST treatment (**i**) compared with controls (**f**), *n*=5. Statistical analyses were performed using a multiple comparison Student’s *t*-test with unequal variances comparing *dye,* 0.1% DMSO groups to TST-treated larvae, ***P<0.001, **P<0.01 *P<0.05

To determine the effect of TST on visual impairment *in vivo* we utilized the *dye*^*ucd6*^ zebrafish model of inherited blindness, identified in a forward genetics screen for visual function mutants and characterized by defects in visual behavior and retinal morphology.^36^ First, the effect of TST on *dye* larvae was analyzed by immunoblot procedure. *dye* larvae were treated with 100 *μ*M TST from 3 to 5 days post fertilization (dpf), larval eyes (50 per each condition) were collected and lysates were subjected to immunoblot analysis. The results show that *in vivo* TST treatment resulted in an increase of acetylated α-tubulin when compared with *α*-tubulin, suggesting that TST successfully inhibits HDAC6 in *dye* zebrafish (Figure 5a). Next, visual behavior and retinal morphology was assessed. TST induced a 16.42-fold increase in eye saccades, as measured by the optokinetic response (OKR) assay, when compared with vehicle (0.1% DMSO)-treated control *dye* larvae (Figure 5b). The visualmotor response (VMR) assay quantifies locomotor activity in direct response to changes in light conditions. In response to 100 *μ*M TST treatment, *dye* larvae showed a 2.52- or 2.07-fold increase in the MAX OFF or MAX ON VMR, respectively, compared with vehicle (0.1% DMSO)-treated control *dye* larvae (Figure 5c). In agreement, TST treatment improved retinal morphology in *dye* as evidenced by a 77.94% reduction in the number of dying cells with pyknotic nuclei (red boxes in Figures 5d and g, insets e and h, quantification j) in the peripheral retina or ciliary marginal zone (CMZ) and a qualitative improvement in the appearance of photoreceptors (Figures 5f and i). In summary, TST treatment improved two cone photoreceptor-mediated visual behaviors in a zebrafish model of inherited retinal degeneration.

## Discussion

The tubulin deacetylase HDAC6 is considered as a stress surveillance factor, is a key player in neurodegenerative diseases and a potential target for neuroprotection and regeneration. It is involved in a variety of cellular events, including proteasome dysfunction, cell adhesion regulation, heat-shock responses and redox regulation.^21, 23, 24, 37^ The overexpression of HDAC6 has been connected to neurodegenerative disorders, and its specific suppression may provide protection.^38, 39^ Here we show that HDAC6 is constitutively expressed in the murine retina and may play a role in retinal diseases, which involve oxidative damage and photoreceptor cell death. Using cone-like 661W cells our data demonstrate that in response to oxidative stress and HDAC6 inhibition by the specific inhibitor TST, cell survival was promoted and the levels of HSP25 and HSP70 were elevated. Furthermore, TST influenced the redox regulatory protein Prx1, which is a target of HDAC6 and displays antioxidant capacity.^13, 40^ Also, in the *dye* zebrafish model of inherited sight loss we demonstrate that TST was sufficient to rescue cone-mediated visual function and improve retinal morphology.

Previous studies using mouse models of RP and cone dystrophies have implicated that pan-inhibition of HDACs (class I and II) prevented photoreceptor cell death.^18, 20^ However, general HDAC inhibitors lead to the deacetylation of histones and thus influence gene transcription. Additionally, they increase the acetylation of many non-histone proteins.^41, 42^ Hence, the finding that HDAC6 in the retina is involved in neuroprotection, and that its specific inhibition has beneficial effects, opens up new therapeutic possibilities using isoform-specific inhibitors. HDAC6 does not act on histones and its major substrate is *α*-tubulin. Acetylated *α*-tubulin occurs on polymerized microtubules (MT) and affects MT dynamics and stability by slowing down the rate of MT growth and shrinkage.^43^ Furthermore, *α*–tubulin acetylation has been demonstrated to be involved in cell adhesion processes. In this respect it was shown that hypoacetylation caused a reduction in cell to substrate adhesion,^44^ and that a failure to deacetylate *α*-tubulin after HDAC6 inhibition resulted in increased cell adhesion and a decrease in cell motility.^45^ Hence, a decrease in MT dynamics after HDAC6 inhibition in cells may promote cell adhesion properties, which might additionally contribute to the preserved cell morphology and cell survival mechanisms, as observed in 661W cells in the present study. In this context it is of interest to note that a delay in developmental migration or a misplacement of cone photoreceptors is a common feature of retinal degenerative diseases, and that HDAC inhibition by the pan-inhibitor Trichostatin A improved cone migration in a mouse model of inherited primary cone degeneration.^20^

As mentioned above, HDAC6 is involved in a heat-shock response, and HSP90 was identified as its substrate. HSP90 is a molecular chaperone that either stabilizes or promotes the degradation of its client proteins, depending on its acetylation status, which is mediated by acetyltransferase p300 and HDAC6.^46, 47^ Hyperacetylation of HSP90, for example, due to HDAC6 inhibition, decreases its affinity for ATP, impairs its chaperone function and favors client degradation.^46^ Furthermore, inhibition of HSP90 exerts neuroprotective effects by upregulation of HSPs in retinal degenerative disorders that are associated with defects in proteolytic systems.^15, 48^ Here we show that treatment of 661W cells with TST caused an increase in HSP70 and HSP25 by activation of HSF1. HSF1 activity is essential for the expression of many HSPs, including HSP25 and HSP70. In unstressed cells HSF1 is present in a repressive complex involving HDAC6, p97/VCP and HSP90. In response to stress the complex dissociates, HSF1 is activated and translocates to the nucleus where it binds to promoter regions of heat-shock genes triggering their expression.^27^ Especially HSP25 and HSP70 exert important roles in the retina by protecting cells against oxidative stress and even retinal degeneration.^16, 17^ Incubation of 661W cells with KRIBB11, a specific inhibitor of HSF1 activation, repressed the induction of HSP70 and HSP25 under the condition of oxidative stress. However, this did not attenuate the protective effect of TST against H_2_O_2_-induced cytotoxicity as determined by an MTT cell viability assay. This points to the conclusion that the beneficial effects exerted by these HSPs are not sufficient to prevent cell damage in the present settings and other factors contribute.

The data presented in Figure 4 suggest that peroxiredoxin 1 (Prx1) might be involved in the protective action of TST. Prx1 is a member of the peroxiredoxin family, a class of antioxidant proteins that catalyze the reduction of peroxides and protect cells against oxidative stress.^40, 49^ The peroxide reducing activity of Prx1 is dependent on post-translational modifications, in particular acetylation, which is mediated by HDAC6.^25, 49^ The depletion of HDAC6 activity, either by siRNA-mediated knockdown or inhibition by tubacin, which represents a less specific derivative of TST,^28^ resulted in hyperacetylated Prx1.^25^ Thus, Prx1 is a specific target of HDAC6 and acetylation increases its reducing activity and resistance to superoxidation. Peroxiredoxins have multiple functions, and besides being sensors for redox signals, they are involved in the regulation of diverse cellular processes, including cell growth, metabolism, and cell death and survival. They are upregulated in cancer and inflammatory diseases, and may provide protection in neurodegenerative diseases that are associated with oxidative damage.^40^ The expression of peroxiredoxins in various compartments of the human eye, and their special abundance in the retina and iris, has been reported. Alterations in their expression patterns after pathological UV-B-associated processes and oxidative-type DNA damage was observed.^50^ Furthermore, peroxiredoxins were prominently expressed in the retina and optic nerve of rats, and specifically localized in neurons of the INL and cone photoreceptors.^51^ Hence, peroxiredoxins may be crucial in protecting retinal cells and in particular photoreceptors, which are exposed to a high level of ROS, from oxidative stress-induced injuries.

To determine the effect of TST treatment on visual degeneration *in vivo* the *dye* zebrafish model of inherited sight loss was utilized. The mutation in *dye* affects the *atp6v0e1* gene, a subunit of the vacuolar ATPase complex.^36^ This complex is involved in phagocytosis and acidification of intracellular organelles including lysosomes.^52^ The mutant is characterized by cell death in the CMZ and defects in photoreceptor morphology, leading to severely reduced visual function. These defects may be explained by an inability of retinal pigment epithelial cells to degrade discarded photoreceptor outer segments.^53^ As photoreceptor OS accumulate ROS due to high metabolic activity and exposure to light,^54^ an inability to degrade these components may lead to excessive levels of ROS in the retina. Visual function can be readily assayed in larval zebrafish by behavioral assays,^55, 56^ and as rod photoreceptor-mediated vision does not develop until later stages,^57^ the responses recorded in this study are mediated by cone photoreceptors. As mentioned previously, class I/II HDAC inhibitors (HDACi) demonstrate neuroprotective and restorative effects on retinal morphology in mouse models of retinal degeneration.^20, 58^ While isoform selectivity of HDACi is a contentious issue, trichostatin A and scriptaid, two pan- or class I/II selective HDACi have potent inhibitory activity against HDAC6.^59^ Thus, HDAC6 inhibition may have a significant role in the neuroprotective effects of these less selective HDACi. In addition to the likely accumulation of ROS in the retina, this suggests that the protective effect of TST in *dye* may be mediated via regulation of peroxiredoxin activity.

## Materials and Methods

### Materials and antibodies

Cell culture media were obtained from Invitrogen (Grand Island, NY, USA). Tubastatin A hydrochloride (TST) was purchased from Sigma-Aldrich (Munich, Germany), KRIBB11 was from Merck Millipore (Darmstadt, Germany) and H_2_O_2_ from Roth (Karlsruhe, Germany). MTT (1-(4,5-dimethylthiazol-2-yl)-3,5-diphenylformazan) was obtained from USB Corporation (Cleveland, OH, USA). For immunoblot analysis the following antibodies were used: mouse monoclonal antibody (mAb) anti-*α*-tubulin (T9026), 1:5000 and mouse mAb anti-acetylated *α*-tubulin (T6793), 1:1000, were from Sigma-Aldrich. Goat pAb anti-HDAC6 (sc-5258), 1:500, was purchased from Santa Cruz (Heidelberg, Germany). Rat mAb anti-HSF1 (ADI-SPA-950), 1:500, was from Enzo Life Sciences (Lörrach, Germany). Rabbit pAb anti-HSP25 (ADI-SPA-801), 1:2000, and rabbit pAb anti-HSP70 (ADI-SPA-812), 1:1000, were both from Enzo Life Sciences. Rabbit pAb anti-peroxiredoxin 1 (ab15571), 1:1000, and rabbit pAb anti-peroxiredoxinSO_2/3_ (ab16830), 1:10 000, anti-*α*-tubulin (ab76449), 1:2000 dilution for western blot of zebrafish protein lysate, were obtained from Abcam (Cambridge, UK). HRP-conjugated anti-mouse IgG (1:10 000) and anti-rabbit IgG (1:10 000) were from Jackson ImmunoResearch (West Grove, PA, USA). Indirect immunofluorescence on 661W cells was performed using mouse mAb anti-acetylated *α*-tubulin (Sigma-Aldrich, T6793), 1:250, secondary antibody goat anti-mouse Dylight 488 (Thermo Fisher Scientific, Waltham, MA, USA, 35502), Phalloidin-CF594 (Biotium, Hayward, CA, USA, 00045), 1:100 and 4′,6-diamidino-2-phenylindole dihydrochloride (DAPI) for nuclear staining, 100 ng/ml (Sigma-Aldrich, D9542).

### Animals

For the experiments 3- to 5-months old wild-type C57BL/6J mice (Charles River, Wilmington, MA, USA) were used. Mice were maintained on a 12/12 h light/dark cycle with food and water *ad libitum*. Mice were handled and killed in accordance with the institutional guidelines for animal welfare at the University of Oldenburg and the laws on animal experimentation issued by the German government (Tierschutzgesetz). Mice were anesthetized with CO_2_ and killed by cervical dislocation. Eyes were enucleated in physiological phosphate-buffered saline (PBS; pH 7.4) and the cornea, lens and vitreous were removed from the eyecup. For RT-PCR retinas were dissected from the eyecup in RNAse-free conditions and immediately frozen in liquid nitrogen. For immunoblot analysis retinas were homogenized in sample buffer containing 2% SDS.

All experiments utilizing zebrafish were performed in accordance with approval granted by the UCD Animal Research Ethics Committee. Homozygous *dye* larvae were obtained from natural spawning of heterozygous adults. Larvae were maintained on a 14 h light/10 h dark cycle at 28.5 °C in embryo medium (0.137 M NaCl, 5.4 mM KCl, 5.5 mM Na_2_HPO_4_, 0.44 mM KH_2_PO_4_, 1.3 mM CaCl_2_, 1.0 mM MgSO_4_ and 4.2 mM NaHCO_3_, conductivity 1200 *μ*s, pH 7.2) until 3 days post fertilization (dpf).

### Cell culture and treatment

In this study the immortal mouse photoreceptor-derived 661W cell line, obtained from Dr. Muayyad Al-Ubaidi (Department of Cell Biology, University of Oklahoma, Health Sciences Centre, Oklahoma City, OK, USA), was used. 661W cells were kept in DMEM supplemented with 10% fetal bovine serum, 2 mM glutamine, 50 U/ml penicillin and 50 *μ*g/ml streptomycin. Cell morphology was monitored by Hoffman modulation contrast imaging (Olympus IX70/SF8, Tokyo, Japan). To analyze the effect of HDAC6 inhibition, oxidative stress and HSF1 inhibition on protein expression in 661W cells, 350 000 cells were seeded on 10 cm cell culture dishes (TPP, Trasadingen, Switzerland) and allowed to attach and proliferate for 48 h. Cells were incubated with TST, H_2_O_2_ and KRIBB11 as indicated. To evaluate the effect of TST in response to oxidative stress, 661W cells were either left untreated or were incubated with 1, 5 and 10 *μ*M TST for 8 h, with 200 *μ*M H_2_O_2_ for 6 h or preincubated with 1, 5 and 10 *μ*M TST for 2 h followed by 200 *μ*M H_2_O_2_ for 6 h in the presence of TST. To study whether TST induces HSF1, 661W cells were treated with 10 *μ*M TST for 1, 3 and 6 h, or with 5 *μ*M KRIBB11 for 6.5 h, or preincubated with KRIBB11 for 30 min followed by TST treatment in the presence of KRIBB11 for further 1, 3 and 6 h. After treatment, cells were washed with PBS and either lysed in sample buffer containing 2% SDS for immunoblot analysis or subjected to indirect immunofluorescence. TST and KRIBB11 stock solutions were prepared using DMSO. H_2_O_2_ was diluted in aqua bidest before treatment.

### Indirect immunofluorescence

661W cells were cultured on glass coverslips (Fisher Scientific, Schwerte, Germany) for 48 h in DMEM/10% FBS/P/S and incubated with TST for 24 h. After washing with PBS, cells were fixed with 3% paraformaldehyde (PFA) for 15 min and permeabilized with 0.1% Triton-X-100 for 20 min. After another washing step, unspecific binding sites were blocked with 5% BSA for 30 min. Cells were incubated with anti-acetylated *α*-tubulin antibody, overnight at 4 °C and washed three times with PBS. Incubation with secondary antibody and Phalloidin-CF594 was conducted for 1 h at room temperature in the dark, cells were washed and mounted. Nuclei were stained by 4′,6-diamidino-2-phenylindole (DAPI; 1.5 *μ*g/ml) included in the mounting medium (Vectashield; Vector Laboratories, Burlingame, CA, USA).

### Fluorescent image acquisition

Fluorescent staining of 661W cells was analyzed using a Leica TCS SL confocal microscope (SP8, Wetzlar, Germany). Scanning was performed with an oil-immersion × 63/NA1.40 objective. Confocal stacks were processed with ImageJ (NIH, Bethesda, MD, USA). Brightness and contrast were adjusted using Adobe Photoshop CC 2015 (San Jose, CA, USA).

### Immunoblot analysis

661W cells, mouse and zebrafish retina lysates were boiled for 10 min and protein concentrations were determined according to Neuhoff *et al.*^60^ For immunoblotting, total cellular extracts (10–40 *μ*g protein per lane) were separated by one-dimensional SDS polyacrylamide gel electrophoresis (SDS-PAGE) using 7.5 and 10% polyacrylamide gels and transferred onto nitrocellulose membranes (Whatman, Dassel, Germany; 0.2 *μ*m). Unspecific binding sites were blocked with 5% powdered milk in TBS (20 mM Tris, 137 mM NaCl, pH 7.5), for peroxiredoxin antibodies blocking was performed using RotiBlock (Carl Roth). Blots were incubated with primary antibodies overnight at 4 °C. After washing with TBS with 0.1% v/v Tween 20 (TBS-T), incubation with HRP-conjugated anti-mouse (1:10 000) or anti-rabbit (1:10 000) antibody was carried out for 1 h at RT. Membranes were washed again several times with TBS-T and signals were visualized by the enhanced chemiluminescence procedure as described by the manufacturer (Thermo Scientific, Rockford, IL, USA).

### RNA extraction and RT-PCR

RNA from C57BL/6J retina lysates was isolated using a RNA preparation kit (NucleoSpin RNA XS, Macherey Nagel) following the manufacturer's instruction. Additional DNAse digestion was performed with DNaseI Amp Grade (Invitrogen) using 1 *μ*g of RNA. Complementary DNA (cDNA) was synthesized for 50 min at 50 °C in a final volume of 20 *μ*l. Each sample contained 25 *μ*g/ml oligo-(dT) 15 primer and 25 *μ*g/ml random primer (Promega, Madison, WI, USA), 0.5 mM dNTP (Carl Roth). After primer annealing (5 min at 65 °C), samples were cooled on ice prior to the addition of 2 U/*μ*l RiboLock (Thermo Fisher Scientific), 1 × first-strand buffer, 5 mM DTT and 10 U/*μ*l of SuperScriptIII reverse transcriptase (Invitrogen, Carlsbad, CA, USA). RNA from untreated 661W cells was extracted using the RNeasy Mini Kit according to the manufacturer's protocol for animal cells (Qiagen, Hilden, Germany). One microgram of RNA was used for reverse transcription in a final volume of 25 *μ*l. First-strand synthesis was performed with 2 mM of each dNTP, 0.6 *μ*M each of oligo-(dT)18 (Fermentas, Fisher Scientific, Schwerte, Germany) and random hexamer primer (Promega), 4 *μ*l of 5x M-MLV-reaction buffer, 40 U of recombinant RNasin ribonuclear inhibitor and 200 U of M-MLV reverse transcriptase (Promega). After a denaturation step (70 °C for 5 min) and incubation at 37 °C for 1 h, 40 *μ*l of DEPC-treated water was added to the reaction mixture. One microliter of cDNA was used for PCR analysis.

PCR reactions were carried in a volume of 25 *μ*l containing DreamTaq reaction buffer, 0.025 U/*μ*l DreamTaq DNA polymerase, 0.2 mM of each dNTP (Thermo Fisher Scientific) and 0.4 *μ*M of each primer: HDAC6 (intron spanning), 5′-CTGCAGTCGCTATGTCAATG-3′ and 5′-GTCCTCCCCAAACTTGTTCT-3′ (Biomol, Hamburg, Germany) and GAPDH, 5′-TGGCAAAGTGGAGATTGTTG-3′ and 5′- ACTGTGGTCATGAGCCCTTC-3′ (synthesized by Invitrogen, Darmstadt, Germany). For HDAC6 detection 27 cycles, consisting of 30 s denaturation at 94 °C, 30 s primer annealing at 60 °C and 45 s elongation at 72 °C, were run in a BiometraThermocycler. GAPDH was detected by performing 24 cycles, consisting of 30 s denaturation at 94 °C, 30 s primer annealing at 62 °C and 45 s elongation at 72 °C. After a final extension for 4 min at 72 °C samples were stored at 4 °C. Eight microliters were used for analysis by agarose gel electrophoresis.

### MTT assay

To assess the influence of KRIBB11 on the cell viability of TST- and H_2_O_2_-treated 661W cells, the colorimetric MTT (tetrazolium) assay was carried out. 661W cells were plated on 96-microwell cell culture plates (4500 cells/well) and allowed to adhere and proliferate for 24 h in 100 *μ*l DMEM/10% FBS/P/S. Growth medium was replaced with 100 *μ*l of fresh medium only or medium supplemented with 10 *μ*M TST, 5 *μ*M KRIBB11 and 200 *μ*M H_2_O_2_, respectively. Cells were incubated with TST for 8 h, with KRIBB11 for 8.5 h and with H_2_O_2_ for 6 h. Furthermore, cells were preincubated with TST for 2 h followed by H_2_O_2_ for 6 h in the presence of TST, or with KRIBB11 for 30 min followed by TST for 2 h followed by H_2_O_2_ for 6 h in the presence of KRIBB11 and TST. Ten microliters of MTT solution (5 mg/ml in PBS) was added and cells were incubated for 2 h. Thereafter, 100 *μ*l of solubilization solution (10% sodium dodecyl sulfate in 0.01 mol/l HCl) was added and incubated overnight to dissolve the water-insoluble formazan salt. Quantification was carried out using an ELISA reader for measuring absorbance at 595 nm. Data are expressed as the percentage of the untreated control, which was set at 100% and values represent the mean±S.D. of eight microwells of one experiment. Three independent experiments were performed with similar results concerning the protective effect of TST and the influence of KRIBB11. The high variability of H_2_O_2_ cytotoxicity, however, led to the exclusion of these data from statistical analysis. One representative result is depicted in Figure 4.

### Larval zebrafish drug treatment

Three dpf *dye* larvae and controls were placed in embryo medium and incubated with drug dissolved in DMSO, to a final concentration of 100 *μ*M TST and 0.1% DMSO or 0.1% DMSO alone. Larvae were maintained on a 14 h light/10 h dark cycle at 28.5 °C until 5 dpf when removed from drug solution and used for immunoblotting, histology and visual function assays.

For immunoblot analysis 5 dpf TST or 0.1% DMSO treated *dye* larvae were killed via anesthetic overdose (4% Tricaine), transferred to a protein extraction buffer (50 mM HEPES/KOH pH 7.2, 5 mM EGTA, 10 mM KCl, 2 mM MgCl2, 0.2% SDS) and enucleated. Fifty larval eyes for each condition were collected, were sonicated at 5% amplitude for 10 s using a Soni-prep 150 (Sanyo) in 50 *μ*l extraction buffer and sample buffer containing SDS was added. Cell lysates were subjected to immunoblot analysis as described above.

For histology 5 dpf larvae were killed with a lethal dose of tricaine. Whole larvae were fixed in 4% PFA and 2.5% glutaraldehyde in 0.1M Sorensons’s buffer (pH 7.3) overnight at 4 °C. Samples were post-fixed using 1% osmium tetraoxide, dehydrated in ascending concentrations of ethanol and transferred to descending propylene oxide:Epon 812 resin mixtures before embedding in EPON resin alone. Embedded samples were polymerized overnight at 60 ºC. One micrometer sections were prepared using a Leica EM UC6 microtome and glass knife, mounted on glass slides and stained with toluidine blue. Sections were coverslipped and imaged using a Leica DMLB bright field illumination microscope and Leica DFC 480 camera × 100 objective. Whole-eye images are composites from at least two images; insets are digitally zoomed images of regions of interest. The number of dying cells were quantified by counting the number of pyknotic nuclei present in multiple sections in the central retina (10 *μ*m distance apart).

### OKR assay

Individual 5 dpf larva were immobilized in a Petri dish using 9% methylcellulose solution. The Petri dish was placed inside a circular 18º repeating black and white striped pattern at 99% contrast.^55^ The pattern was rotated around the immobilized larva at 18 revolutions per minute for 30 s in both clockwise and anti-clockwise directions, and the number of saccadic eye movements produced by the larva per minute was recorded.

### VMR assay

The VMR assay quantifies changes in locomotor behavior of larvae in response to transitions in light conditions. Twelve larvae per treatment group were transferred in 600 *μ*l embryo medium to separate wells of a transparent 650 *μ*l 96 square-well plate. The plate was transferred to the Zebrabox recording chamber (Viewpoint Life Sciences, Montreal, QC, Canada), and larvae were allowed to acclimate to light conditions for 1 h before recording is initiated. During the 1 h 40 min recording period there is a 20 min period in light, followed immediately by 20 min in the dark, these cycles were repeated until the recording period was complete. Detection settings and analysis were carried out as previously described.^61^ Average MAX ON and average MAX OFF responses were determined by the average maximum locomotor activity (measured in ms/s) per treatment group in the 5 s period after a transition to light or dark conditions.

### Statistics

All experiments were carried out at least three times with similar results, unless stated otherwise. Quantitative evaluation of immunoblots was performed by densitometric analysis using ImageJ. For HSP25 and HSP70 analysis protein levels were normalized to actin and expressed as relative to the control, which was set as 1. For PrxSO_2/3_ quantification values were normalized to Prx1 protein bands and expressed relative to H_2_O_2_-treated cells, set as 1. Statistical significance was determined using a two-sided paired *t*-test (IBM SPSS Statistics, Version 24). Normal distribution of differences was tested (Shapiro Wilk test) and significance levels were *P*<0.05 (*), *P*<0.01 (**) and *P*<0.001 (***). For zebrafish visual function and retinal morphology data were analyzed using an independent two-sample *t*-test with unequal variances.
