# Supplementary material for: HDAC6 inhibition by tubastatin A is protective against oxidative stress in a photoreceptor cell line and restores visual function in a zebrafish model of inherited blindness
Source: Cell Death Dis. 2017 Aug 31;8(8):e3028–. doi: 10.1038/cddis.2017.415 (PMC5596594; doi:10.1038/cddis.2017.415)
Supplement: Supplementary information [file cddis2017415x1.docx]

**HDAC6 inhibition by tubastatin A is protective against oxidative stress in a photoreceptor cell line and restores visual function in a zebrafish model for inherited blindness**

Janina Leyk^1^, Conor Daly^2^, Ulrike Janssen-Bienhold^3^, Breandán Kennedy^2^ and Christiane Richter-Landsberg^1^

^1^Department of Neuroscience, Molecular Neurobiology, University of Oldenburg, D-26111 Oldenburg, Germany

^2^School of Biomolecular and Biomedical Science, Conway Institute, University College Dublin, Belfield, Dublin, D04 V1W8, Ireland.

^3^Department of Neuroscience, Visual Neuroscience, University of Oldenburg, D-26111 Oldenburg, Germany

**Supplementary information**

To test the specificity of the HDAC6 antibody used, cell lysates from C57BL/6J mice retinae and 661W cells were prepared and subjected to Western blot procedure. HDAC6 antibodies were incubated with the corresponding blocking peptide. Immunoblot analysis revealed that the blocking peptide led to the removal of HDAC6 specific bands.


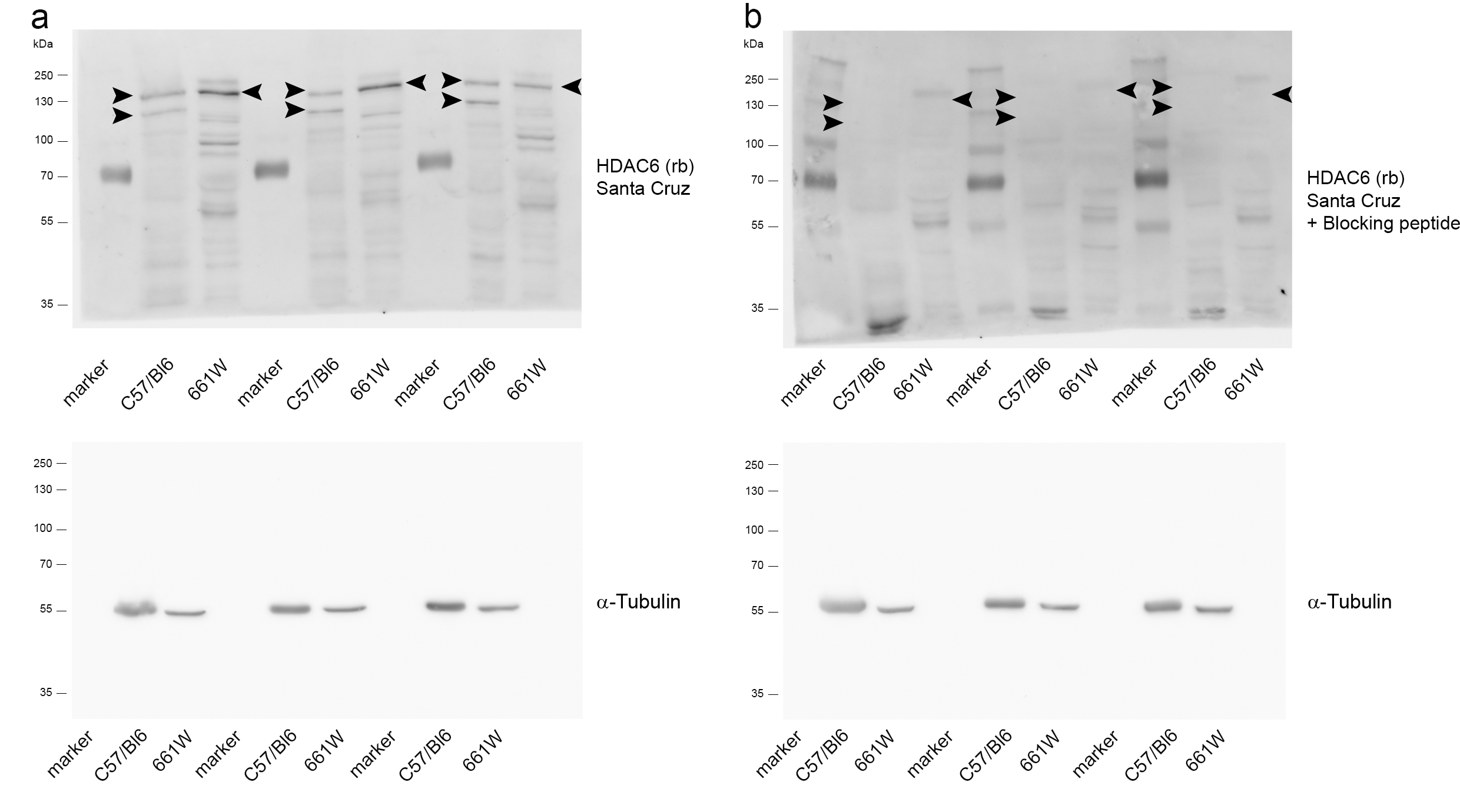


Figure 1: Supplementary information to Figure 1 of the manuscript. Cell lysates were prepared from three independently collected samples of C57BL/6J mice retinae and 661W cells and subjected to immunoblot analysis. To block the antibody´s specific binding sites, HDAC6 antibody (Santa Cruz, sc-5258) was incubated with the according blocking peptide (Santa Cruz, sc-5258 P) in a ratio of 1:10 in PBS for 4h at room temperature. Thereafter, immunoblot analysis was conducted using control (a) and blocked (b) antibody. Arrow heads indicate a major HDAC6 peptide with an apparent molecular weight of 134 kDa, present in both cell lysates, and a 114 kDa present in C57BL/6J mice retinal cell lysates (a), which both were removed after antibody blocking (b). Subsequent to HDAC6 immunoblotting, membranes were incubated with anti-α-Tubulin antibodies as a loading control.
